# Clinic at the Harlem Hospital

**Published:** 1893-05

**Authors:** Thomas H. Manley

**Affiliations:** New York


### CLINIC AT THE HARLEM HOSPITAL APRIL 19, 1893.

BY DR. THOMAS H. MANLEY.

### RECTOPEXY, PHLEBECTOMY FOR VARICOSE-VIENS IN THE LEG, AMPUTATION ABOVE THE KNEE-JOINT, OSTEOTOMY FOR GENU-VALGUM.

Dr. Manly remarked that the first case was one of an unusual pathological character. He said: this patient, a lady of 52 years, a private patient, consented to come to the clinic, to-day, in order that he might exhibit her case and demonstrate the method which he usually pursues in this class of cases.

She is suffering from what may, with perfect propriety, be designated rectal-hernia, of the relapsing type, a condition erroneously known as procedentia-ani.

She comes to us now, after an interval of four years. She first came under my care four years ago, after different surgeons in various places had tried in vain different experiments to effect relief. She had worn a rectal truss, had the parts seared with the actual cautery, and other things, but all to no avail

My treatment consisted in completely excising the entire extended mass; in doing practically a rectopexy; or resection of the bowel. She enjoyed perfect health until four months ago, when she commenced to suffer from a feeling of constant uneasiness in the rectum. When at stool, after emptying the bowel, she will yet have a lingering desire to strain further. There is, in fact, a pulling in of the bowel. There is no pain and no soreness. Different procedures have been devised in this condition. One of the most radical has been that recommended by Le Dentu of doing a laparotomy, raising the slack of the rectum, from within, and anchoring it to the abdominal wall.

But this is a dangerous procedure, which cannot be justified except in extreme cases.

In this instance the sphincter will be widely dilated, the bowel swollen out and the superfluous mass raised, after which, the chasm will be solidly closed in by silk and cat-gut stitches, applied in thin layers.

The operator said that the procedure was a bloody one if full haemostatic measures were not employed.

The operation was proceeded with and consumed nearly one hour in its performance.

The next case was an extensive varix of the internal saphenous vein. This man, contrary to the usual rule, gave no history of the disease coming through heredity. He was a stone-cutter, had never severely injured his limb, or had any very serious internal disease.

This varix gave him no annoyance unless he stood or walked ; when, he had almost unbearable sense of painful lameness in the limb. Most every sort of relief measure had been resorted to in vain.

On examination we at once came on to an enlarged, tortuous gristly mass, the remnants of the once healthy vein. Now, it is evident that the valves had all given way, and that calcareous changes had begun in the connective tissue laminae of the vessels' wall

We propose to resect the entire diseased mass; cutting upward until we come on to healthy vascular tissue. Properly performed, this is a safe, simple and radical measure.

The operation was performed in the usual manner.

The next case was one of amputation above the knee joint. Dr. Manley said that as this case had been present previously and the questions of tuberculosis, excision and amputation, had been discussed, he would say nothing now except on technique and the circumstances which require us to adapt special operative manipulations for special cases. This young woman of twenty years of age, is very weak and has a high temperature, pulse 150, temperature 103½. An amputation is resorted to only as a last recourse.

In this operation there are two things of cardinal importance to be observed : first, to amputate quickly, and secondly, to spare the loss of blood. In pathological cases we can adapt an amputation to special requirements. In this case we will do the circular method, as it is the quickest, and I believe on the whole, leaves the best stump. We will employ no antiseptic solutions.

Everything being in readiness, the severance of the limb and securing of the arteries was but the work of a moment.

The fourth case was one of double genu-valgum. Patient was but five years old. Dr. Manley said that with these cases, where deflection was in the femora, an osteotomy was done through the convexity, taking care to avoid the epiphyseal line and the main blood trunks. Then the bone should be only nicked with the osteotome, when, by osteoclasis, we complete the work of rectification by an incomplete periostial fracture. The house surgeon, Dr. Arch Dixon, Jr., performed the operation.

